# Optic nerve avulsion: Case report

**DOI:** 10.1016/j.amsu.2021.102554

**Published:** 2021-07-08

**Authors:** Ahmed Mahjoub, Ilhem Sellem, Anis Mahjoub, Nadia Ben Abdesslem, Mohamed Ghorbel, Hachmi Mahjoub, Leila Knani, Fethi Krifa

**Affiliations:** Farhat Hached University Hospital, Sousse, Tunisia

**Keywords:** Optic nerve avulsion, Blunt ocular trauma, Traumatic optic neuropathy, Case report

## Abstract

**Introduction:**

Optic nerve avulsion is a traumatic disinsertion of optic nerve fibres from the globe at the level of the lamina cribrosa. It is an uncommon and severe complication of blunt ocular trauma.

**Case presentation:**

We report the case of a 15 years old male presented to the emergency department after being kicked by a horse. Initial ophthalmologic examination of the left eye (LE), exhibited eyelid hematoma, subconjunctival hemorrhage, VA was limited to light perception and there was a left relative afferent pupillary defect. Dilated fundus examination of the LE revealed an extensive vitreous and preretinal hemorrhage overlaying the optic disc and retina edema.The diagnosis of LE optic nerve head avulsion (ONA) was made. Five years after the accident, VA of LE detecting hand motion, fundus examination revealed a superior dragging of the optic disc, fibroglial scarring, retinal vessel narrowing and retinal epithelium hyperplasia.

**Clinical discussion:**

In case of ONA, the avulsion can be missed initially due to vitreous and retinal hemorrhage overlaying the optic nerve, in such cases multimodal imaging can be a useful tool to the diagnosis and to evaluate associated ocular damage. Healing process of the avulsed optic nerve is characterized by the development of fibroglial proliferation. Visual outcome is poor and the final visual acuity range from light perception or no light perception in total ONA.

**Conclusion:**

Optic head nerve avulsion is a rare and severe disease and initial diagnosis is challenging due to associated media opacities. The prognosis is poor and the injury leads to permanent visual impairment.

## Highlights

• Optic nerve avulsion is rare and serious complication of blunt ocular trauma.
• Domestic, work related and sport related accidents are incriminated.
• Diagnosis is challenging because of media opacities associated.
• The prognosis is poor.
• To date, there is no efficient treatment.

## 1 Introduction

Optic nerve avulsion is a traumatic disinsertion of optic nerve fibers from the globe at the level of the lamina cribrosa [1]. It is an uncommon and severe complication of blunt ocular trauma. The diagnosis can be initially challenging due to the association with media opacities such as vitreous hemorrhage [2]. Multimodal imaging can be a useful tool to the diagnosis and to evaluate associated ocular damage.

This case report has been reported in line with the SCARE Criteria [3].

## 2 Case presentation

**Fig. 1:**
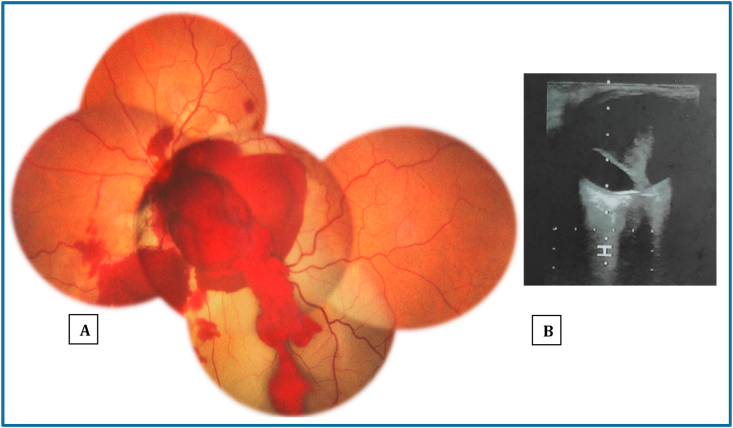
Initial examination. A: Fundus photography reveals an extensive vitreous and preretinal hemorrhage overlaying the optic disc and retina edema. B: Ocular ultrasound shows posterior vitreous detachment.

A 15 years old male with no past medical history, presented to the emergency department for oculofacial trauma after being kicked by a horse. Initial ophthalmologic examination of right eye (RE) showed eyelid hematoma, visual acuity (VA) was 10/10 in Snellen chart, the rest of examination including dilated ocular fundus was normal. Examination of the left eye (LE), exhibited eyelid hematoma, subconjunctival hemorrhage, VA was limited to light perception and there was a left relative afferent pupillary defect. Dilated fundus examination of the LE revealed an extensive vitreous and preretinal hemorrhage overlaying the optic disc and retina edema (Fig. 1 A). Ocular ultrasound revealed posterior vitreous detachment (Fig. 1 B) and computed tomography of the head and orbit showed bilateral lamina papyracea fracture.

The diagnosis of LE optic nerve head avulsion was made and the patient was admitted for hospital surveillance for 10 days, there was no improvement of the final VA at the time of discharge.

**Fig. 2:**
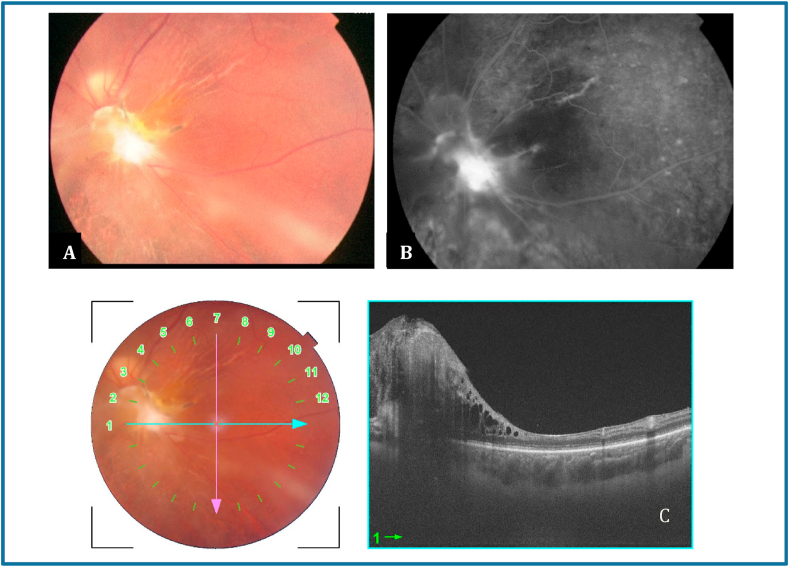
Control examination six months after the injury. A: Fundus photography revealing A fibroglial scarring in the optic disc area, some retinal pigment epithelium hyperplasia and narrowing of retinal vessels. B: FA revealing hyperfluorescence of the fibroglial scarring tissue. C: SS OCT exhibiting the fibroglial scarring as well as marked thinning of the macula.

Six months later, LE VA was detecting hand motion, fundus examination revealed fibroglial scarring in the optic disc area, some retinal pigment epithelium hyperplasia and narrowing of retinal vessels (Fig. 2 A). Fluorescein angiography (FA) showed hyperfluorescence of the fibroglial tissue (Fig. 2 B). Ss OCT exhibited the fibroglial scarring as well as a marked thinning of the macula (Fig. 2 C).

**Fig. 3:**
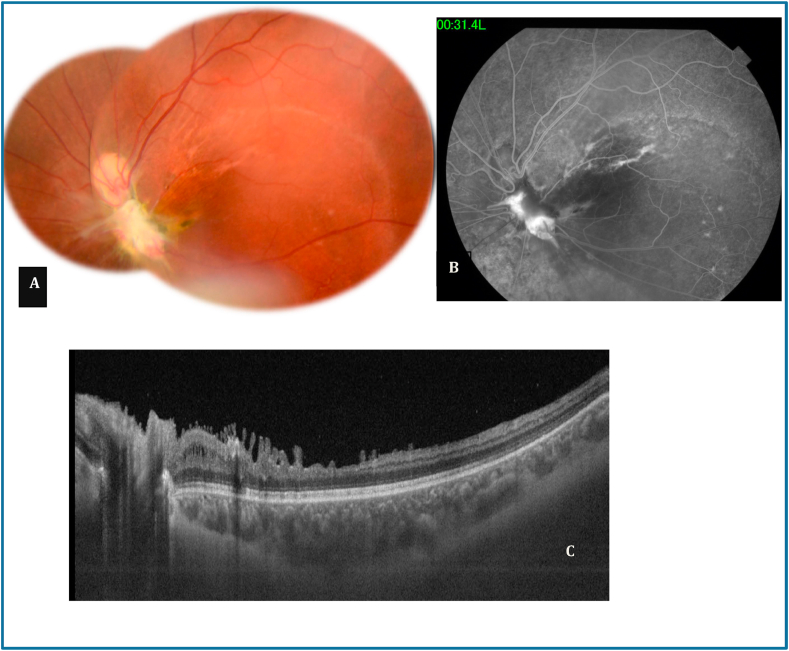
Five years after the injury. A: Fundus photography shows a superior dragging of the optic disc, fibroglial scarring, retinal vessel narrowing and retinal epithelium hyperplasia. B: FA shows hyperfluorescence of the cicatricial tissue. C: SS OCT shows disorganisation of internal retinal layers and macular thinning.

Five years after the accident, VA of the RE had remained 10/10 and on the LE, VA was detecting hand motion, fundus examination revealed a superior dragging of the optic disc, fibroglial scarring, retinal vessel narrowing and retinal epithelium hyperplasia (Fig. 3 A). FA showed hyperfluorescence of the cicatricial tissue (Fig. 3 B). SS OCT exhibited disorganisation of internal retinal layers and macular thinning (Fig. 3 C).

This case report has been reported in line with the SCARE Criteria [3].

## 3 Discussion

Optic nerve avulsion (ONA) is a rare and severe ocular injury which occurs after a setting of a rapid rotational force applied to the globe [1,4]. The avulsion consists of a disinsertion of the optic nerve from the eye and the lamina cribrosa from the scleral rim. The avulsed optic nerve is either partially or completely disinserted and it can be associated with other retinal lesions [1,5]. Several mechanisms have been proposed such as severe forced rotation of the globe which avulse the optic nerve from a weaken scleral area, the second theory is that a sudden rise in the intraocular pressure caused by compression of the globe or by sudden forward propulsion of the globe as a result of increased intraorbital pressure causing disruption at the weaker lamina cribrosa [6]. It was classically reported after a finger poke to the globe [7] but it has also been reported after bicycle or motorcycle accident and animal kick [8]. The avulsion can be missed initially due to vitreous and retinal hemorrhage overlaying the optic nerve, in such cases, ocular ultrasonography (US) can be helpful and may exhibit a posterior ocular wall defect in the region of the optic nerve head characterized by hypoechoic defect [2]. In our case, ocular US does not reveal signs of ONA. After the resorption of the vitreous and preretinal hemorrhage, the diagnosis of ONA by fundus examination become easier and it shows an excavation in the optic disc area [9].

Healing process of the avulsed optic nerve is characterized by the development of fibroglial proliferation [10]. Visual outcome is poor and the final visual acuity range from light perception or no light perception in total ONA [11]. As to the management, procedures that have been proposed were optic nerve fenestration, corticosteroid and watchful surveillance but there was no difference between different procedures in term of final VA [10].

## 4 Conclusion

Optic head nerve avulsion is a rare and severe disease and initial diagnosis is challenging due to associated media opacities. To date, there is no efficient treatment. The prognosis is poor and the injury leads to permanent visual acuity impairment. Prevention of ocular trauma is the most efficient management.

## Ethical approval

Not applicable.

## Please state any sources of funding for your research

No source of funding.

## Consent

Written informed consent was obtained from the patient and from his legal guardians (parents) for publication of this case report and accompanying images. A copy of the written consent is available for review by the Editor-in-Chief of this journal on request.

## Author contribution

All authors made a significant contribution to this paper.

## Registration of research studies

Name of the registry:
Unique Identifying number or registration ID:
Hyperlink to your specific registration (must be publicly accessible and will be checked):

## Guarantor

Guarantor: Ilhem Sellem M.D.

E-mail: Ilhem.sellem@gmail.com.

## Declaration of competing interest

The authors declare no conflict of interest.
